# Identification of differentially expressed genomic repeats in primary hepatocellular carcinoma and their potential links to biological processes and survival

**DOI:** 10.3906/biy-2104-13

**Published:** 2021-10-18

**Authors:** Gökhan KARAKÜLAH, Cihangir YANDIM

**Affiliations:** 1 İzmir Biomedicine and Genome Center (İBG), İzmir Turkey; 2 İzmir International Biomedicine and Genome Institute (İBG-İzmir), Dokuz Eylül University, İzmir Turkey; 3 Department of Genetics and Bioengineering, Faculty of Engineering, İzmir University of Economics, İzmir Turkey

**Keywords:** Liver cancer, hepatocellular carcinoma, satellite RNA, transposable elements, retroelements, RNA sequencing

## Abstract

Hepatocellular carcinoma (HCC) is one of the deadliest cancers. Research on HCC so far primarily focused on genes and provided limited information on genomic repeats, which constitute more than half of the human genome and contribute to genomic stability. In line with this, repeat dysregulation was significantly shown to be pathological in various cancers and other diseases. In this study, we aimed to determine the full repeat expression profile of HCC for the first time. We utilised two independent RNA-seq datasets obtained from primary HCC tumours with matched normal tissues of 20 and 17 HCC patients, respectively. We quantified repeat expressions and analysed their differential expression. We also identified repeats that are cooperatively expressed with genes by constructing a gene coexpression network. Our results indicated that HCC tumours in both datasets harbour 24 differentially expressed repeats and even more elements were coexpressed with genes involved in various metabolic pathways. We discovered that two L1 elements (L1M3b, L1M3de) were downregulated and a handful of HERV subfamily repeats (HERV-Fc1-int, HERV3-int, HERVE_a-int, HERVK11D-int, HERVK14C-int, HERVL18-int) were upregulated with the exception of HERV1_LTRc, which was downregulated. Various LTR elements (LTR32, LTR9, LTR4, LTR52-int, LTR70) and MER elements (MER11C, MER11D, MER57C1, MER9a1, MER74C) were implicated along with few other subtypes including Charlie12, MLT2A2, Tigger15a, Tigger 17b. The only satellite repeat differentially expressed in both datasets was GSATII, whose expression was upregulated in 33 (>90%) out of 37 patients. Notably, GSATII expression correlated with HCC survival genes. Elements discovered here promise future studies to be considered for biomarker and HCC therapy research. The coexpression pattern of the GSATII satellite with HCC survival genes and the fact that it has been upregulated in the vast majority of patients make this repeat particularly stand out for HCC.

## 1. Introduction

Primary liver cancer is one of the most prevalent cancers, holding the top second place among cancer-related mortalities (Wong et al., 2017). Despite the major improvements in oncology, the prognosis of this devastating disease remains poor. Further understanding of underlying molecular and physiological factors and exploiting them for therapeutic purposes could help to overcome this situation. While it is certain that the molecular complexity of liver and the multiple cell types in this tissue adds to the phenomenon, primary liver cancers are almost always from the hepatocyte origin (Tummala et al., 2017). 

Hepatocellular carcinoma (HCC) has often been linked to an underlying liver condition such as fat deposition, steatosis or fibrosis as well as alcohol use and HPV/HCV infections (Llovet et al., 2016). From the molecular perspective, various influential pathways are involved. These include p53 and Rb pathways and other master cell cycle regulators. Also, signalling pathways including TGF-β, Wnt/β-catenin, Notch, Ras/MAPK and PI3K/AKT pathways were reported (Llovet et al., 2016). All of the events leading to hepatocarcinogenesis and resistance to therapy are undoubtedly projected from the genomic plasticity/instability and epigenetic dysregulation in cancerous liver cells (Niu et al., 2016; Toh et al., 2019). 

Aberrant patterns of DNA methylation as well as expression changes and mutations were observed in HCC for a significant number of epigenetic factors. Such pathological changes on the fabric of chromatin are thought to impair the genomic architecture, giving rise to plastic alterations in hepatocellular characteristics (Fernández-Barrena et al., 2020). A plastic genome is unstable and hence more suitable for molecular evolution throughout the initial and metastatic stages of cancer. While many genes are influenced by this instability, it is highly likely that this is the result of a more generalised phenomenon, where chromatin is affected globally. To uncover and comprehend such global effects, sequences outside the genes should as well be studied elaborately. Among such sequences, repetitive DNA comes across as the predominant portion. Even though most of the genomic studies disregard the repeats, they actually make up almost half of the human genome (Richard et al., 2008). The dysregulation of these elements was not elucidated in many types of cancers including hepatocellular carcinoma, where this study focuses on. 

The human repeatome consists of more than a thousand types of repeat motifs, which include the satellites and transposons, including long interspersed nuclear elements (LINEs), short interspersed nuclear elements (SINEs), long terminal repeat (LTR) and DNA transposons. Specifically, satellites are well known for their functions in maintaining chromatin integrity and nuclear architecture by acting as the de novo triggers of heterochromatin (Probst et al., 2010). On the other hand; transposons which are thought to be the evolutionary remnants of ancient virus infections are known to contribute to gene regulation by acting as chromatin modulatory units (Branco and Chuong, 2020). Importantly, the dynamics of repeat expression is well regulated during human embryonic development (Yandim and Karakulah, 2019) and is also associated with cellular senescence (De Cecco et al., 2019). It is noteworthy though, most of the repeats are normally expressed only at basal levels in a healthy human cell (Iglesias and Moazed, 2017). 

An interesting discovery was made in pancreatic cancer and various other epithelial origin cancers, where the satellite repeats HSATI (Zhu et al., 2011) and HSATII (Ting et al., 2011) were reported to be explicitly upregulated in the tumour tissue and contribute to genomic catastrophes by various molecular mechanisms (Zhu et al., 2011; Bersani et al., 2015; Kishikawa et al., 2016). In addition to these, many transposons were reported to be dysregulated in cancer (Burns, 2017). Hence, the therapeutic potential of targeting the repeatome is now well-recognised (Ishak et al., 2018). Also, the potential of repeat-arisen transcripts to serve as cancer biomarkers is being explored with promising results. For example, transcripts arisen from pericentromeric HSATII satellite DNA are known to be highly-enriched in the blood of pancreatic cancer patients and have the potential to serve as biomarker (Kishikawa et al., 2016). 

Despite the emerging role of genomic repeats in various cancers, their contribution to HCC transcriptome remains still elusive. Limited studies reported that simple microsatellite repeats with small repeat motifs (less than 10 nucleotides) exhibited unstable genomic lengths (on DNA) in the HCC tissue (Togni et al., 2009). As for the longer repeats motifs, aberrant DNA methylation patterns were reported for pericentromeric satellites and various other repeats along with LINE (L1) elements (Saito et al., 2001; Anwar et al., 2019; Zheng et al., 2019). Interestingly, methylation patterns of genomic repeats (specifically LINE- L1 family) are known to be influenced by hepatitis virus infections (HBV and HCV) (Honda, 2016; Zheng et al., 2019), resulting in the activation of repeat originated promoters in genes (Bard-Chapeau et al., 2014; Hashimoto et al., 2015). In line with this, jumping transposons and resultant insertions are now considered as a mutagenic force for the evolution of HCC (Schauer et al., 2018). 

Though previous studies pointed out certain repeat classes, the identities of differentially expressed individual repeat subtypes in HCC have not been elucidated yet in a holistic transcriptome analysis. Also, none of such studies checked classical satellite repeats within this concept. Importantly, repeat and noncoding RNA quantification is challenging in comparison to genes (Treangen and Salzberg, 2011) and unsuitable RNA-seq data could jeopardise the findings (Solovyov et al., 2018). In this study, we addressed these issues by employing two independent and publicly available Gene Expression Omnibus (GEO) RNA-sequencing HCC datasets, which were both previously published to be suitable for noncoding RNA and repeat quantification ( Yang et al., 2017; Wu et al., 2020). We collected matched normal and primary tumour tissue RNA-seq data from 20 patients in the GSE77509 dataset (Yang et al., 2017), and from 17 patients in the GSE101432 dataset (Li et al., 2019). We analysed the differential repeat expression profile of both datasets in liver tumour tissues in comparison to their matched normal liver tissue and determined 24 common repeats, half of which were upregulated and the other half downregulated. Additionally, we performed a weighted gene coexpression analysis (WGCNA) and identified common Gene Ontology (GO) terms in both datasets where repeats appeared in correlation with modules of protein-coding genes. The pericentromeric repeat GSATII stood out in our analyses and interestingly it showed significant correlation with HCC survival genes. 

## 2. Materials and methods

### 2.1. Transcriptome data acquisition and processing

Raw sequencing reads of both datasets were extracted from the Sequence Read Archive database (Leinonen, et al. 2011) (SRA Accessions: SRP069212 and SRP111914) with the SRA Tool Kit (v.2.9.0), using “fastq-dump -gzip -skip-technical -readids -dumpbase -clip -split-3” command. We only used data from primary tumours and disregarded relapse tumours or those with portal vein thrombosis.

The human reference genome GRCh38 (hg38) and its reference annotation (release 34) in gene transfer format (GTF) were collected from the GENCODE project website.^1^GENCODE (2020). [online]. Website https://www.gencodegenes.org [accessed 02.11.2020]. Repetitive DNA annotation associated with GRCh38 reference genome was downloaded from RepeatMasker. Repetitive DNA annotation associated with GRCh38 reference genome was downloaded from RepeatMasker.^2^RepeatMasker (2020). [online]. Website http://www.repeatmasker.org/ [accessed 02.11.2020].The sequencing reads of both datasets were aligned to the human reference genome with the R-package Rsubread (v1.34.7) (Liao et al., 2019) using the following command: align(index={index file}, readfile1={input_1.fastq}, readfile2={input 2.fastq} type= “rna”). To sort and index all BAM files produced in the alignment step, we utilised SAMtools (v1.3.1) suite, commonly used for handling high-throughput sequencing data (Li et al., 2009).

The featureCounts function of the Rsubread package was used for the quantification of repeat expressions as well as GENCODE-annotated genes (Liao et al., 2014). In this analysis step, we utilised the following command: featureCounts(files = {infile. bam}, annot.ext = “{infile.gtf}”, isGTFAnnotationFile = T, GTF.featureType = “exon”, GTF.attrType = “gene_id”, useMetaFeatures = T, countMultiMappingReads = T, isPairedEnd = T). We removed repeat element features that overlapped with exonic regions of GENCODE-annotated genes from the annotation file to increase the accuracy of estimated repeat expressions. Only uniquely mapped sequencing reads aligned to DNA, LINE, SINE, LTR, and satellite repeat regions were considered, and repeat element and GENCODE-annotated gene counts were merged into a single expression matrix for downstream analysis.

### 2.2. Differential expression analysis of repeat elements and statistical metaanalysis of HCC data sets

We computed counts per million (CPM) values for each repeat element and GENCODE-annotated gene across all samples in both datasets. In order to increase detection sensitivity of differentially expressed repeat features, we removed all features with mean expression values less than one CPM in normal and tumour conditions. To find differentially expressed repeat features between normal and tumour for each dataset, the EdgeR package v3.24.3 of the R environment was used (Robinson et al., 2010). Trimmed mean of M-values (TMM) normalisation (Robinson and Oshlack, 2010) was applied to count values, and dispersions were estimated with estimateDisp function for each comparison. To calculate the false discovery rate (FDR) of each repeat feature, we made use of exactTest function of edgeR.

For the meta-analysis of HCC datasets, we used Fisher p-value combination and inverse normal p-value combination methods (Hernandez-Segura et al., 2017). To apply these methods, we made use of fishercomb and invnorm functions of the R-package metaRNaseq (v1.0.3) (Rau et al., 2014). Repeat elements with a combined p-value ≤ 0.01 in both methods and absolute log_2_(fold change) ≥ 0.6 were considered as significant.

### 2.3. Weighted gene coexpression analysis (WGCNA) of protein-coding genes and repeat elements followed by module preservation analysis

We used the R-package WGCNA (v1.47) (Langfelder and Horvath, 2008) to construct individual coexpression networks for both HCC transcriptome datasets. Each correlation network was created by calculating correlations between all genomic features including repeat and protein-coding genes across samples. CPM values of features were used as input. The soft threshold value of the correlation matrix was selected as 12 and average linkage hierarchical clustering method was used for grouping the genes with similar expression patterns. To determine network modules, we used the dynamic tree cut algorithm (Langfelder et al., 2008) and minimum module size was designated as 30 genes. Next, we determined module eigengene values by calculating the first principal component of each module separately. 

In order to discover preserved network modules between two independent HCC datasets, we used modulePreservation function of WGCNA package with default parameters. The GSE77509 dataset was employed as the reference set while the GSE101432 expression data was used as the test set. Thus, we validated the network modules found in the GSE77509 data. We calculated the medianRank and Zsummary statistics of module preservation and number of permutations parameter was set to 200 times in this step.

### 2.4. Statistical analysis and graphical representation

We employed R (v4.0.2) programming language^3^The R Foundation (2020). The R Project for Statistical Computing [online]. Website https://www.r-project.org/ [accessed 02.11.2020].  for all statistical computing and graphics in the study. GO enrichment analyses of WGCNA coexpression modules were performed with the clusterProfiler (v3.18.0) (Yu et al., 2012) package of the R environment, and the *cor.test* function was used for the calculation of Pearson correlation coefficients and the significance levels. Other graphics were obtained using the ggplot2 (v3.3.2) package (Wickham, 2016). 

## 3. Results

### 3.1. Global profile of repeat expression in HCC

**Figure 1:**
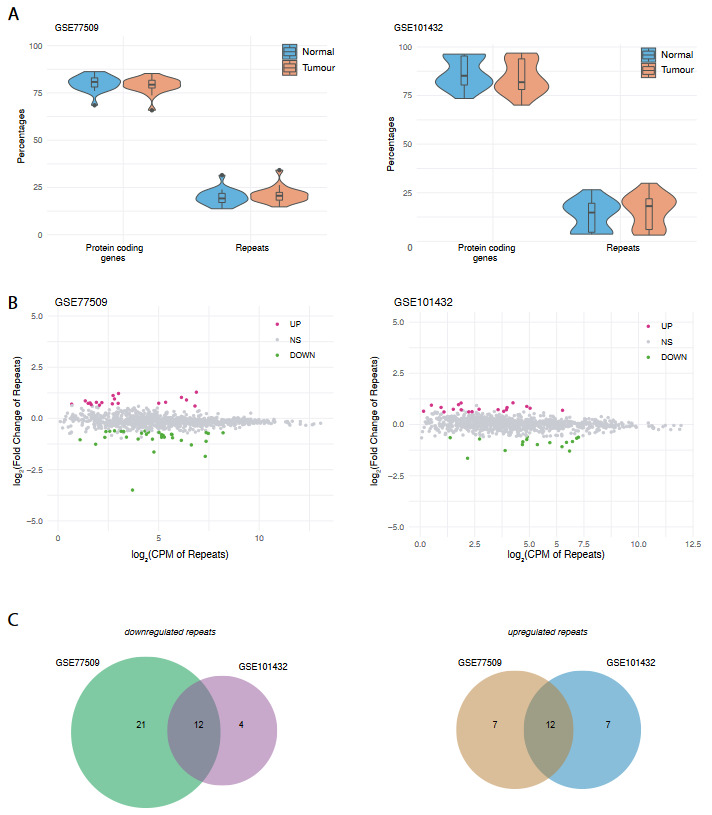
Metaanalysis of differentially expressed repeats in HCC tumour vs. matched normal tissues in two independent datasets (GSE77509 and GSE101432). (A) Violin plots representing the distribution of transcripts. (B) MA plots indicating upregulated (UP), downregulated (DOWN) repeats and other nonsignificant (NS) repeats. (C) Venn diagrams indicating down- and upregulated repeats in both datasets (a filter of |log2(fold change) |≥ 0.6 and combined p-value <0.01 was applied.).

In order to compare the repeat expression profiles in the tumour and matched normal tissue, we calculated the CPM values for each individual gene and repeat – separately for both HCC datasets. Next, we converted the expression values to read percentages, where the maximum CPM values were presented as 100% (Conesa et al., 2016). The distributions of read percentages were slightly different both for genes and repeats – albeit not statistically significant (Figure 1A). A slight increase in the global expression profile of repeats was noticed in the tumour tissues in comparison to matched normal. The MA plots, which help to visualise the distribution of differential expression (McDermaid et al., 2019), revealed upregulated and downregulated repeats for each HCC dataset (Figure 1B). Next, we wanted to identify the genomic repeats that were differentially expressed in both datasets. After applying a differential expression analysis, where we performed Fisher p-value combination and inverse normal p-value combination methods for both datasets (Hernandez-Segura et al., 2017), we realised 12 repeats were downregulated and 12 of them were upregulated in both datasets with a combined adjusted p-value less than 0.01 and absolute log_2_(fold change) threshold greater than or equal to 0.6 (Figure 1C). 

### 3.2. Individual genomic repeats differentially expressed in HCC

**Figure 2:**
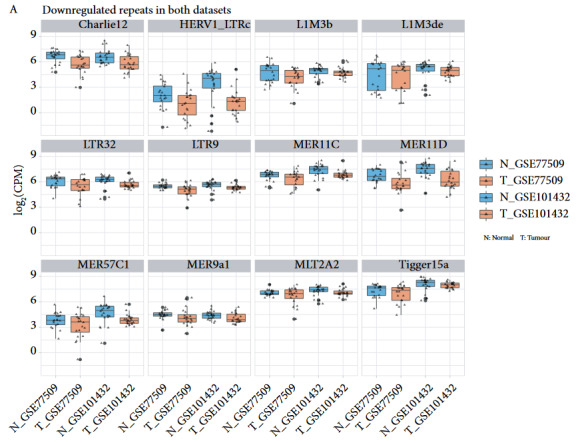

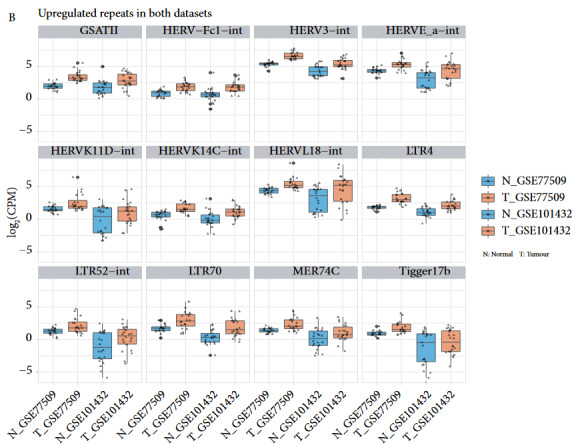
Box and whisker plots for consistently downregulated (A), and upregulated (B) repeat elements in both HCC datasets. All of these repeats were found to be differentially expressed in a statistically significant (combined p < 0.01) manner in both datasets. Triangles represent individual data points.

We plotted the raw CPM values of statistically significant 12 downregulated and 12 upregulated repeats in both datasets separately and realised that some of them displayed higher variation among patients along with outliers (Figure 2). This could indicate that subgroups of patients display a more pronounced effect. Among the repeats that were differentially expressed in both independent HCC datasets, there were some DNA and LTR transposons and LINE elements (Table 1). We were not able to detect any SINE elements. L1 family members of LINE elements only came up as downregulated. Members of the HERV/HERVK subfamily of the ERV1 LTR transposons only came up among the upregulated repeats. Various particular LTR elements and DNA transposons were also detected. Interestingly, there was one satellite repeat that came upregulated; the pericentromeric repeat GSATII. Some of the repeats in this list were previously mentioned in cancer literature, and some of them were novel as discussed later.

**Table 1:** Genomic repeats that were differentially expressed in both HCC datasets.

Repeat name	Repeat family	Repeat class	log2 (fold change) GSE77509	log2 (fold change) GSE10432	Fisher-combined p-value	Inverse-normal-combined p-value
GSATII	centr	Satellite	1.2196	0.7492	6.36907E-11	1.72882E-09
LTR4	ERV1	LTR	1.1205	0.7178	1.01284E-08	5.00577E-08
HERV3-int	ERV1	LTR	1.0284	0.7978	3.78805E-15	4.06385E-15
LTR70	ERV1	LTR	0.9535	1.0513	0.000130102	7.18428E-05
HERVK14C-int	ERVK	LTR	0.8582	0.8363	5.30701E-06	4.43727E-06
HERVL18-int	ERVL	LTR	0.7911	0.8835	0.000441129	0.000441129
MER74C	ERVL	LTR	0.7724	0.6144	0.001072908	0.000779251
HERVE_a-int	ERV1	LTR	0.7444	1.0580	2.15513E-05	2.51666E-05
HERV-Fc1-int	ERV1	LTR	0.7400	0.9694	2.02374E-05	0.000125362
HERVK11D-int	ERVK	LTR	0.7134	0.7460	0.005541907	0.003559382
Tigger17b	TcMar-Tigger	DNA	0.6751	0.6499	0.003213935	0.00213086
LTR52-int	ERVL	LTR	0.6487	0.9452	0.001411917	0.000863754
L1M3b	L1	LINE	–0.6799	–0.8455	1.43746E-09	6.09163E-10
MLT2A2	ERVL	LTR	–0.6928	–0.8314	1.72498E-11	1.49777E-11
Tigger15a	TcMar-Tigger	DNA	–0.7015	–0.6200	4.22716E-13	1.66187E-12
LTR9	ERV1	LTR	–0.7692	–0.9815	4.31236E-12	1.6477E-12
MER9a1	ERVK	LTR	–0.7718	–0.9807	6.23882E-08	3.05986E-08
L1M3de	L1	LINE	–0.9121	–0.6030	7.06219E-12	6.67229E-11
LTR32	ERVL	LTR	–1.0702	–0.8866	3.78805E-15	3.78805E-15
MER11C	ERVK	LTR	–1.1146	–0.8702	0	0
Charlie12	hAT-Charlie	DNA	–1.2912	–1.0844	3.42E-09	1.55174E-09
MER57C1	ERV1	LTR	–1.6387	–1.2711	0	0
MER11D	ERVK	LTR	–1.8497	–1.2960	0	0
HERV1_LTRc	ERV1	LTR	–3.4956	–1.6466	0	0

### 3.3. Differentially expressed repeats and their possible contribution to biological functions in HCC

**Figure 3:**
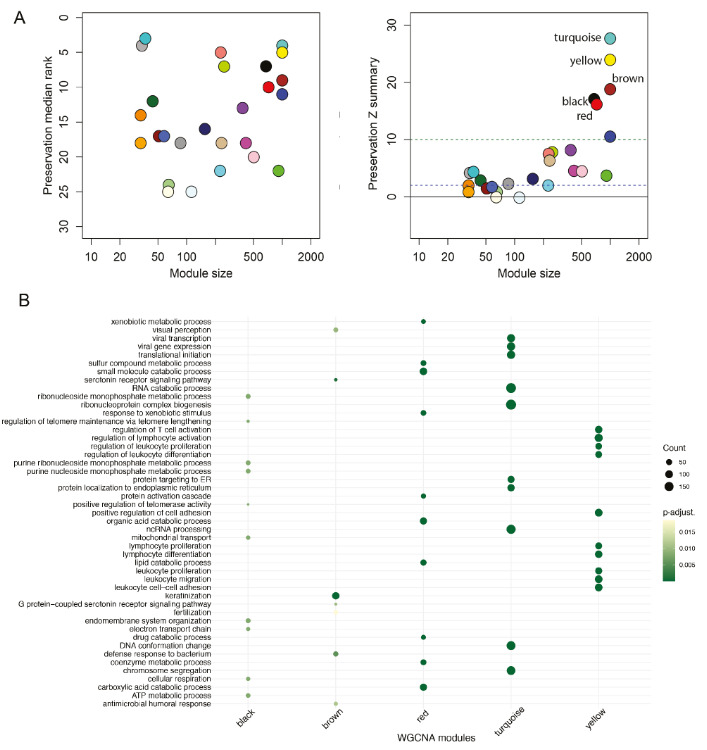
Weighted gene coexpression network analysis (WGCNA) for repeats and genes to reveal their putative biological cooperation. (A) Preserved WGCNA modules shown by their median ranks (left panel) and preservation Z summaries (right panel). (B) Gene ontology analysis revealing biological functions in the preserved WGCNA modules.

Due to reported involvement of repetitive DNA to molecular functions in the cell (Shapiro and von Sternberg, 2005; Yandim and Karakulah, 2019), we aimed to dissect genes, which simultaneously coexpress with repeats so that we could reveal the possible biological functions where repeat dysregulation in HCC could be influential. We performed WGCNA analysis (Zhang and Horvath, 2005) in the pool of repeat- and gene-arisen transcripts, separately for each HCC dataset. This analysis revealed several modules represented with different colours. To highlight the consistency, we identified the preserved modules in both datasets using a previously defined pipeline (Hu et al., 2018). We determined five preserved modules, where repeats were coexpressed with genes (Figure 3A). The repeats falling into each module were given in Table 2. 

WGCNA exposed six differentially expressed repeats (i.e. HERV1_LTRc, LTR32, LTR9, MER11C, MER11D and MER57C1) and many additional elements. Intriguingly, all of the differentially expressed repeats were those that were downregulated in the HCC tissue (Figure 2), and all were detected in the red module. Our GO term analysis on the preserved modules (Figure 3B) brought several biological functions as determined by the coexpressed genes among with repeats. Red module was associated with ribonucleoprotein complex biogenesis; sulphur, drug, coenzyme, lipid and organic acid metabolism/catabolism. On the other hand, turquoise module pointed out viral infection related genes and RNA catabolism, and the yellow module brought out functions involved in lymphocyte differentiation. Brown module was linked to keratinization and the black module was involved in several metabolic pathways including cellular respiration and ATP metabolism. 

**Table 2:** Repeats coexpressed with genes involved in distinct biological functions in preserved WGCNA modules as given in Figure 3. (*) indicates significantly dysregulated repeats.

WGCNA module	Repeat name	Repeat family	Repeat class
black	MER63D	hAT-Blackjack	DNA
black	SAR	Satellite	Satellite
brown	(CATTC)n	Satellite	Satellite
brown	(GAATG)n	Satellite	Satellite
brown	ACRO1	acrocentric	Satellite
brown	CR1-8_Crp	Satellite	LINE
brown	D20S16	Satellite	Satellite
brown	GSAT	centromeric	Satellite
brown	HERV-Fc1_LTR2	ERV1	LTR
brown	HERV-Fc2-int	ERV1	LTR
brown	HERV9-int	ERV1	LTR
brown	HERVFH19-int	ERV1	LTR
brown	HERVFH21-int	ERV1	LTR
brown	HERVH-int	ERV1	LTR
brown	HERVK11-int	ERVK	LTR
brown	HSATI	Satellite	Satellite
brown	L1P4e	L1	LINE
brown	LSAU	Satellite	Satellite
brown	LTR103b_Mam	ERV1	LTR
brown	LTR1C1	ERV1	LTR
brown	LTR1C3	ERV1	LTR
brown	LTR27D	ERV1	LTR
brown	LTR30	ERV1	LTR
brown	LTR46-int	ERV1	LTR
brown	LTR53-int	ERVL	LTR
brown	LTR59	ERV1	LTR
brown	LTR7	ERV1	LTR
brown	LTR72	ERV1	LTR
brown	LTR7A	ERV1	LTR
brown	LTR7C	ERV1	LTR
brown	LTR7Y	ERV1	LTR
brown	LTR9D	ERV1	LTR
brown	MLT1E1-int	ERVL-MaLR	LTR
brown	X1_LINE	CR1	LINE
red	ERV3-16A3_LTR	ERVL	LTR
red	Eulor1	DNA	DNA
red	HERV1_I-int	ERV1	LTR
red	HERV1_LTRc*	ERV1	LTR
red	HERV1_LTRe	ERV1	LTR
red	LTR19-int	ERV1	LTR
WGCNA module	Repeat name	Repeat family	Repeat class
red	LTR22A	ERVK	LTR
red	LTR28	ERV1	LTR
red	LTR32*	ERVL	LTR
red	LTR47A	ERVL	LTR
red	LTR9	ERV1	LTR
red	LTR9A1	ERV1	LTR
red	MamRep1879	hAT-Tip100	DNA
red	MER11C*	ERVK	LTR
red	MER11D*	ERVK	LTR
red	MER44B	TcMar-Tigger	DNA
red	MER57C1*	ERV1	LTR
red	MER84-int	ERV1	LTR
turquoise	AluYe5	Alu	SINE
turquoise	AluYk2	Alu	SINE
turquoise	Charlie10a	hAT-Charlie	DNA
turquoise	HERV1_LTRd	ERV1	LTR
turquoise	HERVIP10B3-int	ERV1	LTR
turquoise	LTR109A2	ERV1	LTR
turquoise	LTR10B1	ERV1	LTR
turquoise	LTR12E	ERV1	LTR
turquoise	LTR6A	ERV1	LTR
turquoise	LTR86B2	ERVL	LTR
turquoise	MSTC-int	ERVL-MaLR	LTR
yellow	AluSx4	Alu	SINE
yellow	LTR21A	ERV1	LTR
yellow	LTR21B	ERV1	LTR
yellow	MST-int	ERVL-MaLR	LTR

### 3.4. GSATII as an emerging satellite repeat in hepatocellular carcinoma

**Figure 4:**
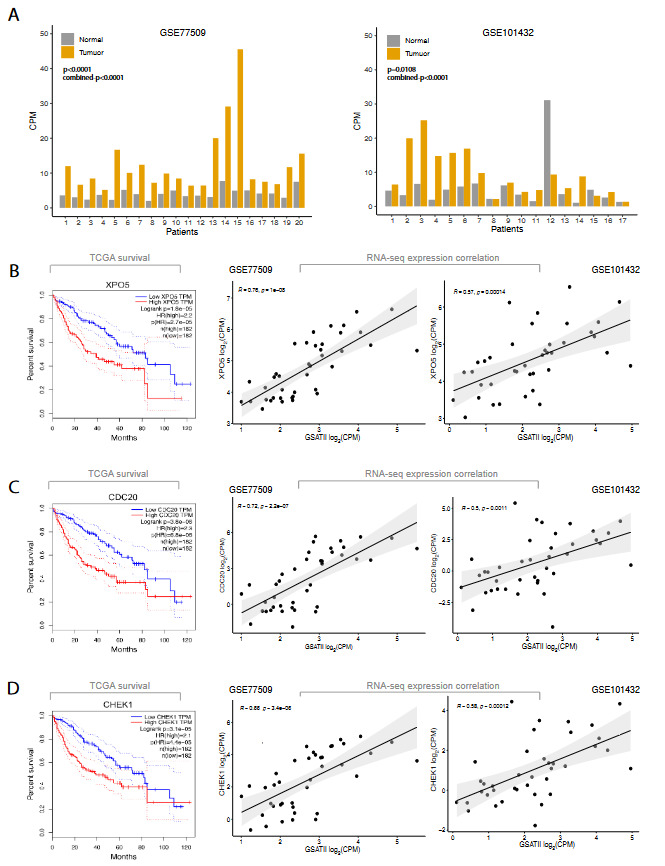
GSATII expression in primary HCC tumours and its correlation with HCC survival genes. (A) Raw GSATII CPM values for individual patients in both HCC datasets. (B-D) Kaplan–Meier survival analyses of TCGA survival genes (left panel) as shown by the GEPIA webtool and their correlations with GSATII in both HCC datasets (middle and right panels).

As introduced above, the degenerative potential of abnormally expressed satellite DNA on the chromatin architecture has been well recognised as a major pathological factor in cancer (Ting et al., 2011; Bersani et al., 2015; Biscotti et al., 2015; Iglesias and Moazed, 2017; Velazquez Camacho et al., 2017). Interestingly, the only satellite repeat (among the 25 members of this repeat class) differentially expressed in the HCC primary tumours was the pericentromeric γ satellite; GSATII, a 216 base pair long tandem repeat according to Repbase (Bao et al., 2015) and DFAM databases (Hubley et al., 2016). GSATII was upregulated in the primary tumours of HCC patients; in all 20 patients in the GSE77509 dataset and in 14 patients out of 17 patients in the GSE101432 dataset (Figure 4A); highlighting this satellite’s upregulation in more than 90% of patients. Next, we checked crucial survival-linked genes in HCC as listed in the GEPIA webtool (Tang et al., 2017). This tool relies on the information of survival and gene expression utilising the HCC dataset of The Cancer Genome Atlas (TCGA 2017) and lists statistically significant survival linked genes based on a log-rank test. Strikingly, 11 out of the top 100 survival-related genes were found to be correlated with GSATII expression in the GSE77509 dataset (Pearson’s r ≥ 0.6). These were given in Table 3. Out of these 11 survival-linked genes, six of them were correlating with GSATII in the GSE101432 dataset as well (Pearson’s r ≥ 0.5). These were CDC20, CHEK1, GPSM2, KIF2C, UCK2 and XPO5. Representative survival and correlation graphs were given for CDC20, CHEK1 and XPO5 (Figure 4B).

**Table 3:** HCC survival genes and Pearson’s correlation scores for GSATII. Significant survival genes were obtained from GEPIA webtool [50].

DatasetSurvival genes	GSE77509	GSE101432
CDC20	0.7152	0.5028
CHEK1	0.6615	0.5767
GARS1	0.6645	0.2194
GPSM2	0.6195	0.5898
KIF2C	0.6723	0.5085
NUP37	0.7258	0.4426
PES1	0.6488	0.2914
PIGU	0.7101	0.3337
UBE2S	0.7021	0.4267
UCK2	0.7082	0.6159
XPO5	0.7632	0.5730

## 4. Discussion

Understanding the molecular phenomena that hepatocellular carcinoma exploits is difficult. The high level of genomic instability reflected by epigenetic events makes the therapy challenging (Fernández-Barrena et al., 2020). Even though the current treatments in clinics focus on multikinase inhibitors (e.g. sorafenib), resistance to therapy emerges easily (Chen and Wang, 2015). In this study, we revealed the complete repeatome dynamics of HCC tumours to shed light on the unknown dimensions of pathological genomic dysfunction. Among more than a thousand repeat motifs, we uncovered 24 differentially expressed elements, which consistently appeared in two independent HCC datasets. 

Our results indicate only one satellite RNA (GSATII) and various LTR, LINE and DNA transposons. The upregulated expression of GSATII could imply the decay of the healthy genomic architecture in HCC as these peri-/centromeric elements are normally not expressed after the first few cell divisions of the human embryonic development (Yandim and Karakulah, 2019), and expressed only at basal levels in the healthy pancreatic tissue with a downregulation in pancreatic adenocarcinoma (Ting et al., 2011). As opposed to other peri-/centromeric repeats, members of the γ-satellite subfamily –where GSATII belongs to– are known to protect nearby gene expression from the invasion of pericentromeric heterochromatin suggesting their insulation activity (Kim et al., 2009). Ikaros and CTCF binding sites are also present on these satellites (Kim et al., 2009). Interestingly, both of these factors were related to HCC (Zhang et al., 2014; Zhang et al., 2017). In addition, another study pointed out GSATII upregulation in blood specimens of nine colon cancer patients (Kondratova et al., 2014) and a statistically insignificant upregulation trend was mentioned in ER+/HER2- primary breast tumours (Yandım and Karakülah, 2019). Our study pointed out an increase in GSATII expression in the majority (> 90%) of HCC patients. The paucity of information on this satellite repeat in literature does not give much room for exploration on the mechanisms; however, future studies on this element within the context of HCC are definitely warranted.

Though the transposon involvement in HCC was reported before (Bard-Chapeau et al., 2014; Hashimoto et al., 2015; Honda, 2016; Schauer et al., 2018; Anwar et al., 2019), to our knowledge this is the first study that outlines the individual subtypes dysregulated in HCC among the overwhelming number of transposons. Dysregulated L1 subtypes L1M3b and L1M3de could be worth being investigated further as L1 family in general was related to patient survival in HCC (Anwar et al., 2019). L1M3b was implicated in splicing, chromatin organisation and organ development in terms of its cooperation with genes during embryonic development (Yandim and Karakulah, 2019). Interestingly, LTR70 transposon that was upregulated in HCC also appeared in the same expression modules with L1M3b in the same study (Yandim and Karakulah, 2019). Another similar element; LTR4 that was upregulated in HCC was also upregulated in lung cancer (Arroyo et al., 2019). Other upregulated repeats that we uncovered included members of the human endogenous retrovirus (HERV) subfamily. Upregulated HERV-FC1-int was reported to be overtly activated in multiple sclerosis (Laska et al., 2012). Moreover, HERVL14C-int upregulation was also reported for breast cancer (Yandım and Karakülah, 2019) and HERV3-int for lung cancer (Arroyo et al., 2019). It is of note that HERV1_LTRc, which was reported to be robustly upregulated in primary breast tumours, was shown to be significantly downregulated in our study for HCC. The latter could be one of the key examples on how genomic repeats behave differently across different cancer types. 

Our analysis on coexpression networks showed that six dysregulated repeats and many other additional repeats act in orchestration with genes highlighting biological pathways. However, contribution of repetitive RNA to cellular function is yet to be figured out. One interesting example could be the sequestering effect of HSATII transcripts on DNA repair proteins (Kishikawa et al., 2016). Given that GSATII structure is highly similar to HSATII (Bersani et al., 2015), such mechanisms could be explored for HCC. GSATII correlation with crucial HCC survival genes in our study suggests the functional importance of this element. On the other hand, whether the rise in GSATII repeat transcripts is indeed due to transcription or due to the expansion of these repeats at the DNA level also remains to be studied further. Expansion of HSATII on DNA was reported for pancreatic cancer (Bersani et al., 2015) and a similar manifestation could be possible for GSATII. 

Given the fact that repeat contents of mouse and human genome differ significantly (Komissarov et al., 2011), biopsy or surgery samples collected from patients are of invaluable use in repeat quantification of the transcriptome. Also, repeats are known to behave pathologically in real tissues and cell lines do not provide the necessary platform for such studies (Ting et al., 2011). Indeed, one challenge prior to our study was to find the datasets suitable for noncoding repeat quantification. Unfortunately large datasets such as those in TCGA were prepared specifically for mRNA transcripts with a poly(A) bias. To assess the genome fully, it is essential to produce sequencing datasets suitable for both coding and noncoding transcripts. Previously mentioned biases mostly were set to save from the expenses but we believe that with the reduction of the costs in sequencing technologies in time, this limitation will be lifted and hence it will be easier to illuminate the unexplored sites of the genome. Despite such challenges, we were still able to confirm our findings in two independent and suitable GEO datasets that comprise primary HCC patient specimens. The functional contribution of dysregulated repeats identified in this study could be illuminated with further research. Moreover, these differentially expressed genomic elements could be targeted for therapy and they also bring the tantalising possibility of serving as a biomarker for disease progress as future studies are warranted.
